# Cardiac biomarkers in the intensive care unit

**DOI:** 10.1186/2110-5820-2-8

**Published:** 2012-03-07

**Authors:** Anthony S McLean, Stephen J Huang

**Affiliations:** 1 Department of Intensive Care Medicine, Nepean Hospital, Sydney Medical School, Penrith, NSW 2750, Australia

## Abstract

Cardiac biomarkers (CB) were first developed for assisting the diagnosis of cardiac events, especially acute myocardial infarction. The discoveries of other CB, the better understanding of cardiac disease process and the advancement in detection technology has pushed the applications of CB beyond the 'diagnosis' boundary. Not only the measurements of CB are more sensitive, the applications have now covered staging of cardiac disease, timing of cardiac events and prognostication. Further, CB have made their way to the intensive care setting where their uses are not just confined to cardiac related areas. With the better understanding of the CB properties, CB can now help detecting various acute processes such as pulmonary embolism, sepsis-related myocardial depression, acute heart failure, renal failure and acute lung injury. This article discusses the properties and the uses of common CB, with special reference to the intensive care setting. The potential utility of "multimarkers" approach and microRNA as the future CB are also briefly discussed.

## Introduction

Blood cardiac biomarkers (CB) have become increasingly accurate for evaluating cardiac abnormalities during the past 40 years. Initially, with the focus on myocardial infarction (MI), the use of creatinine kinase-MB (CK-MB), first described in 1972, was a major step forward in the development of a highly cardiac-specific biomarker. The introduction of cardiac troponin (cTn) assays in 1989 was the next major advance, and subsequent refinement of the assays now has the definition of acute myocardial infarction (AMI) centered on it. This progression ironically has brought considerable difficulties to the critical care physician who deals with multiorgan failure rather than the patient presenting to the emergency department with chest pain or single-organ pathology. The recent penetration of high-sensitivity (hs) cTn, replacing the fourth-generation cTn assays further compounds these diagnostic challenges.

**Figure 1:**
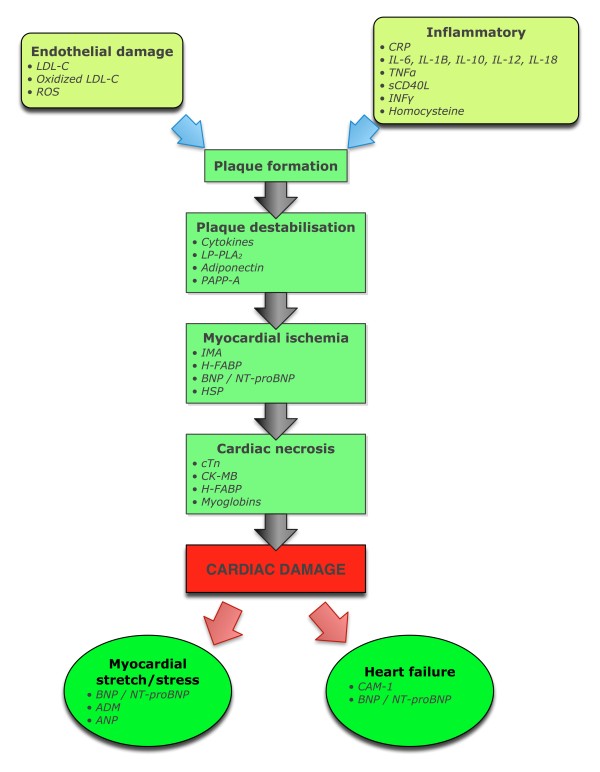
**The development of cardiac biomarkers**. ADM, adrenomedullin; BNP, B-type natriuretic peptide; CAM, cell adhesion molecule; CK-MB, creatine kinase-MB; CRP, C-reactive protein; cTn, cardiac troponin; H-FABP, human fatty-acid binding protein; HSP, heat shock protein; IL, interleukin; IMA, ischemia-modified albumin; INFγ, interferon γ; LP-LPA_2_, lipoprotein-associated phospholipase A_2_; PAPP, pregnancy-associated plasma protein; ROS, reactive oxygen species; sCD40L, soluble CD40 ligand.

Moving beyond a sole focus on MI, the search for alternative and supplementary serum markers to assist in unravelling the presence, severity, and type of cardiac injury has been intense (Figure 1). Whilst cardiac ischemia/infarction is the most prevalent cause of cardiac injury with biomarker development reflecting this, the search for more meaningful biomarkers now includes CB for inflammatory processes and myocardial wall stress (as a result of pressure or volume overload) where evaluation extends beyond myocardial necrosis. The important role of C-reactive protein (CRP) as a prognostic marker is an example of the former while natriuretic peptides are now accepted as clinically useful markers of cardiac stress.

In the critical care setting, the challenge of confounding factors brings about interpretation difficulties. Clarity in diagnosis and/or guidance for management frequently present when the heart is the only organ affected, such as in the emergency department or cardiology ward, does not always hold in the intensive care unit (ICU) setting. Even so, an understanding of the commonly used CB can be very helpful for cardiac evaluation of the critically ill patient.

### Classes of cardiac biomarkers

**Figure 2:**
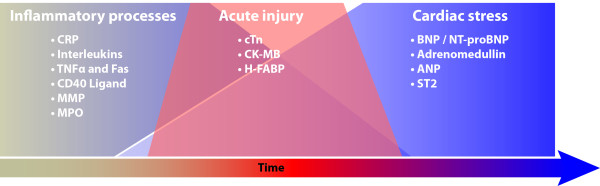
**Evolution of cardiac dysfunction and the associated changes in cardiac biomarkers**. (see legend to Figure 1 for abbreviations).

The search for clinically useful CB has resulted in a large number of circulating plasma substances being investigated. These can be broadly grouped temporally into three major categories: inflammatory, acute muscle injury, and cardiac stress (Figure 2).

#### Biomarkers for inflammatory processes

Cardiac wound healing after MI can be divided into four phases: phase 1 begins with the actual death of myocytes commencing within 6 hours and continuing for up to 4 days; the phase 2 is that of an inflammatory response beginning 12-16 hours after onset of ischemia; phase 3 is when granulation tissue begins forming at the infarct border zone; and phase 4 consists of remodeling and repair and begins at 2-3 weeks, persisting for up to a year [1]. Although a number of immune mediators, including cytokines, autoantibodies to myosin and tropomyosin, as well as interferon (IFN)-γ have been closely studied, clinically useful circulating inflammatory biomarkers to assist in the diagnosis and prognosis of AMI have yet to be established [2].

Where an infective agent is responsible for myocardial damage, acute viral myocarditis being the prototype, once again clinically helpful inflammatory biomarkers are not available. Temporally, three phases exist when myocardial damage is due to infection: 1) myocyte destruction by the virus; 2) an innate immune response, which ultimately may cause more harm than good; and 3) possible myocardial damage, resulting in a dilated cardiomyopathy, and where once again, autoantibodies may play a role [3,4].

What often is overlooked is the role of inflammation in the progression of coronary artery disease (CAD), even in the absence of myocardial necrosis, and here the role of inflammatory markers hold more promise [5]. Unfortunately in the critical care setting, where organs other than the heart are usually compromised, the specificity of these markers, including tumor necrosis factor (TNF)-α, CD-40L, interleukins (IL-18, IL-6, IL-33, and IL-1a), CRP, fibrinogen, pentraxin 3, and matrix metalloproteinases, are markedly compromised. The potential of inflammatory markers is promising however, and even in apparently nonimmune settings, such as emotional stress-induced acute onset cardiomyopathy, inflammatory pathways appear to play a pivotal part [6].

### Biomarkers of myocardial injury

#### Cardiac troponins (cTn)

The evolving story of cTn to diagnose acute myocardial damage is as fascinating as it is beguiling. The troponin era began with the development of a double antibody, two-step enzyme immunoassay for TnT in 1989 [7]. Evolution of these developments now has cTn as the central component of the definition of an AMI [8]. The superior diagnostic power of cTn is demonstrated by correlation with histological findings [9]. A diagnosis of AMI is predicted on the detection of an increase or decrease of cTn, with at least one value > 99% percentile upper reference limit (URL) in patients with evidence of myocardial ischemia. This evolution extended the role of Tn from being a marker only for AMI to that of acute coronary syndrome (ACS) and secondary myocardial damage from conditions, such as pulmonary embolus (PE), cardiac trauma, and chemotherapy-induced myocardial damage. The latest step in the evolution is the development and rapid uptake of the hs-cTn assays around the world.

#### Comparison of cTnT and cTnI

The cTn complex, consisting of three proteins (C, I, and T) encoded by different genes, plays a pivotal role in the modulation of calcium-dependant sarcomere contraction [10,11]. cTnI and cTnT have cardio-specific isoforms not found in skeletal muscle, making for highly specific markers of myocardial damage. Both cTnI and cTnT are released from necrotic myocardium, ischemic and nonischemic-induced, as intact proteins and degradation products [12]. Although experimental data indicates that cTn only leaks out of the cell after cell death, the finding of cTn in marathon runners and after inducible myocardial ischemia, raises the possibility of it occurring in the absence of necrosis [13,14]. The cTnT assay is only available from one manufacturer, whereas cTnI assays are available from a number of vendors, rendering interpretation confusing because of the lack of standardization. It is important that the clinician has an understanding of the assay used in their institution, including analytical quality and limitations. Increasing sophistication of the assays has resulted in fewer false-negatives and false-positives; however, the presence of cTn autoantibodies in the blood or marked hemolysis can produce inaccurate results [15,16].

#### cTn as a diagnostic marker

The central consideration in the interpretation of an elevated serum cTn is that it is a marker of myocardial damage, but on its own it does not determine the etiology of the damage. cTnT and cTnI demonstrate similar diagnostic ability in detection of myocardial damage despite analytical differences [17]. The criteria for diagnosing an AMI are a rising or falling pattern of blood cTn levels in association with clinical features of myocardial ischemia. An international taskforce comprising of the American Heart Association/World Health Foundation/European Society of Cardiology/American College of Cardiology Foundation (AHA/WHF/ESC/ACCF) recommends a cutoff value set at 99^th ^percentile of URL, or the concentration at which the assay achieves a coefficient of variation of 10% if that exceeds the 99^th ^percentile [8,18]. Clinical features of AMI include classical symptoms, ECG changes, regional wall motion abnormalities, or imaging evidence of new loss of viable myocardium. In the absence of these features, an alternative cause of the cTn elevation and myocardial damage should be sought (Table 1).

**Table 1:** Conditions commonly associated with cTn elevations

Arrhythmias*
Aortic dissection*
Acute heart failure*
Coronary vasospasm*
Cardiomyopathy, e.g., postpartum
Coronary vasculitis, e.g., SLE, Kawasaki Syndrome*
Cardiac contusion
Chemotherapy
Hypertension*
Myocarditis
Pulmonary embolus
Sepsis/septic shock
SIRS
Takotsubo cardiomyopathy
Renal failure
Severe neurological disorders
Pulmonary hypertension - severe
Radiofrequency ablation*
Pericarditis
Extreme exertion

*Elevations of cTn in the absence of overt ischemic heart disease or in the patient with normal coronary arteries include those patients with myocardial ischemia from noncoronary disease, and by definition come into the MI type II classification. Certain conditions result in chronic elevations of cTn, including chronic renal failure, chronic heart failure, stable CAD, marked left ventricular wall hypertrophy, and aortic stenosis [11].

The timing of cTn elevations becomes increasingly important with the development of more sensitive assays and an understanding of the manner in which cTn is released from the damaged myocyte is helpful. An acute process involves a rise and fall with an increase occurring 2-4 hours after symptoms and remaining elevated for 7-14 days. Therefore, serial measurements, usually 3-6 hours apart are recommended [19]. The less sensitive cTn assays require significant elevations, whereas the newer hs-cTn assay requires smaller ones. Biological variation may become more important at these lower levels bringing other challenges to interpreting the result [20]. If the initial cTn level is not elevated, then serial measurements over 6-9 hours are necessary. If this second sample is still not elevated but clinical suspicion of an MI remains high, then a further sample at 12-24 hours should be considered. An earlier resampling at 3 hours still provides approximately 80% sensitivity in detecting AMI [21]. Age seems to be a confounding factor. A study that included 1,098 patients who underwent a single hs-cTn sample on presentation to the emergency department with symptoms suggestive of an AMI identified the best cutoff value, separating AMIs from non-AMIs, to be much higher. The hs-cTnT value for patients older than aged 70 years at 54 ng/l was nearly four times the 99^th ^percentile, although close to the 99^th ^percentile for two different hs-cTnI assays [22].

The introduction of hs-cTn, with precision sensitivity improved from the μg/l to the ng/l range, has enhanced the sensitivity and reduced the time to diagnose AMI in the acute setting. The use of a single hs-cTnI measurement at 3 hours in patients presenting with chest pain, using a cutoff of 40 ng/l, gave a negative predictive value (NPV) of 84.1% and positive predictive value (PPV) of 86.7%; these findings predicted a 30% increase in cTn levels at 6 hours [23]. The diagnostic accuracy of a number of different hs-cTn assays were found to be excellent and much superior to the standard assays [24]. Interpretation is reliant upon the baseline level, the increase at a selected time interval (usually 6 hours), and the clinical setting. For example, whereas hs-cTnT assay has a cutoff value of 14 ng/l, a second sample at 6 hours is recommended when the value is between 14-100 ng/l to improve the specificity and PPV of the assay. A single reading above 100 ng/l is considered a high risk for ACS and warrants management accordingly. When testing for the optimal change of cTnI, Apple found a ≥ 30% increase from baseline (admission) level in the 4-10 hours follow-up measurements provided a sensitivity of 71% and specificity of 91% [25]. Giannitsis and associates demonstrated that, compared with the 4^th ^generation cTnT, the admission hs-cTnT assay (at a cutoff of 99^th ^percentile) detected more evolving non-STEMI cases (61.5% vs. 7.7%). The detection by hs-cTnT further improved to 100% within 6 hours. The overall diagnosis of MI increased by 34.6% [26]. A doubling of the hs-cTnT concentration within 3 hours in the presence of a second concentration ≥ 99^th ^percentile is associated with a PPV of 100% and NPV of 88% [26]. Such an approach, or a variant of it, is suitable for the development of protocols in the emergency department but unfortunately less so in the ICU where confounding issues are present.

The sedated hemodynamically unstable patient on positive pressure ventilation does not usually provide the classical symptoms of myocardial ischemia. ECG changes, often on a background of an already abnormal ECG, are nonspecific, and confounding factors, such as renal failure or heart failure, are present. Many conditions commonly found in an ICU are associated with elevated cTn levels, even in the absence of definite coronary artery pathology leading to myocardial ischemia (Table 1). Added to this is the likelihood that many MI will be a type II class as described in the international consensus guidelines (Table 2).

**Table 2:** Different types of myocardial infarction

Types of myocardial infarction	Clinical classification
Type 1	Spontaneous myocardial infarction related to ischemia due to primary coronary event.
Type 2	Myocardial infarction secondary to ischemia due to increased oxygen demand or decreased supply, e.g., coronary artery spasm, coronary embolism, anemia, arrhythmias, hypertension, or hypotension.
Type 3	Sudden, unexpected cardiac death with symptoms and signs of cardiac ischemia. Death occurs before blood cardiac biomarkers able to be measured.
Type 4a	Myocardial infarction associated with percutaneous Intervention.
Type 4b	Myocardial infarction associated with stent thrombosis as documented by angiography or at autopsy.
Type 5	Myocardial infarction associated with coronary artery bypass surgery.

Abridge version based on Reference [8]

The advent of hs-cTn brings a definite improvement in diagnostic accuracy but at the expense of lower specificity, a challenge already encountered on a daily basis in the ICU with standard assays. However, this development should be seen as a positive step with the higher diagnostic accuracy, but further studies in the critically ill population are required to better define its use in this population.

#### Cardiac troponin as a prognostic marker

Elevated cTn is associated with poor prognosis in patients with acute ischemia [27,28]. cTnI and cTnT have comparable prognostic performance, except for patients with chronic renal failure where cTnT but not cTnI was a predictor of a worse outcome [29]. It is noteworthy that the prognostic value of cTn is not limited to ischemic heart disease. In a systematic review of an elevated cTn in critically ill patients, looking at 23 studies involving 4,492 patients, Lim and colleagues found a 2.5 times increased risk of death and an increased length of ICU stay of 3 days and hospital stay of 2.2 days [30].

#### Creatine kinase-MB

When first described in 1972, the electrophoresis methods required for separation of the cardiac isoenzymes had low analytical specificity [31]. An immunoinhibition method resulted in a useful clinical test and creatine kinase-MB (CK-MB), in combination with aspartate transaminase and lactate dehydrogenase, became the triad of biomarkers used for the diagnosis of AMI. CK-MB, is released within 2-4 hours, peaks at 24 hours following pressure overload or ischemia, and returns to normal by 36-72 hours [32]. It is a sensitive marker of MI, but a single measurement on presentation has low sensitivity. Lack of specificity is a problem; 10% of patients experience chest pain with an elevated CK-MB but normal cTn [33]. It is present in small amounts in skeletal muscle (1-3% of total CK), and a large muscle injury can be compounded by the re-expression of proteins producing the CK-MB isoenzyme [34]. The advent of cTn has relegated CK-MB, previously the favorite cardiac biomarker of myocardial necrosis, into the background and its role in the critically ill patient has been superseded.

### Fatty acid binding proteins (FABPs)

Fatty acid binding proteins (FABPs)--small proteins located in the cytoplasm--facilitate transport of fatty acids and other lipids within the cell. Heart-type FABP (H-FABP) is a sensitive marker of myocyte damage and, unlike troponin, is released by both ischemia and necrosis. It also is released more rapidly than troponin, although the advent of hs-cTn will overcome the advantage of FABP in this regard. H-FABP is released from the damaged cell within 1-3 hours, returning to normal by 12-24 hours. It is now available as a point of care test for the diagnosis of AMI in many countries and also has good data to support its use in prognostication from MI [35,36]. The diagnostic sensitivity of H-FABP for cardiac injury is 93.1%, higher than CK-MB and cTn [37]. There is a dearth of information about the use of H-FABP for the diagnosis of MI in the critically ill population; however, there may be a role in the risk stratification of pulmonary embolism (PE) in critically ill patients.

### Biomarkers of cardiac stress

#### Natriuretic peptides (NP)

Primarily for maintaining osmotic and cardiovascular homeostasis, the NP family includes multiple peptides and receptors. Present throughout all vertebrate classes, including fish, amphibians, mammals, and birds, this family of related peptides and specific receptors is an integral system in the control of hydromineral balance and blood pressure [38]. In mammals, the NPs include atrial natriuretic peptide (ANP), brain or B-type natriuretic peptide (BNP), C-type natriuretic peptide (CNP), dendroaspis natriuretic peptide (DNP), and urodilatin. They share similar structures, with ANP, BNP, and CNP having a ring of 17 amino acids, 11 of which are identical. Urodilatin and ANP having an identical ring [39]. The activities of NPs and endothelins (ET) are closely associated, with ANP and BNP inhibiting ET-1 release and ET-1 stimulating NP synthesis [39].

Although the earliest investigations into the application of NP in everyday medicine involved ANP, with more recent investigations on CNP, DNP, and urodilatin, BNP and NT-proBNP is where most effort has been directed. The pre-prohormone BNP is a 134 aminoacid peptide synthesized in ventricular myocytes, with subsequent cleaving into the 108 aminoacid prohormone BNP, which is released into the circulation during hemodynamic stress [40]. Corin, a circulating endoprotease, cleaves the prohormone BNP into the biologically active BNP (32 amino acids) and inactive NT-proBNP (76 amino acids). BNP, having a half-life of 20 minutes, is cleared by cells containing BNP receptors, whereas NT-proBNP, which is cleared by the kidney, has a longer half-life (60-120 minutes); this explains the higher circulating concentrations compared with BNP.

The obvious clinical applications of BNP and NT BNP have led to the development of fully automated assays for both; some understanding by the clinician of the specific assay used is important, because some immunoassays cannot differentiate between active and inactive forms. For example, some assays actually measure a mixture of both BNP and NT-proBNP, whereas various breakdown products of BNP may be included in the assay [41].

##### Clinical application of BNP/NT-proBNP

The clearest clinical benefit of the application of BNP and NT-proBNP has been the diagnosis and prognosis of heart failure. Regular application in the emergency department is based on studies such as **B**reathing **N**ot **P**roperly, where a serum BNP level > 100 pg/ml was demonstrated to diagnose congestive heart failure (CHF) with a sensitivity of 90% and specificity 73% [42,43]. Similar diagnostic accuracy was identified with NT-proBNP, with a level of 500 ng/ml used by most investigators [44,45]. BNP levels can be elevated in patients with CAD, leading to search for a diagnostic and prognostic role in this group [46,47].

Only a weak correlation between pulmonary capillary wedge pressure and serum BNP level in the ICU patient has been found [48]. In a review on the role of BNP and NT-proBNP in the management of heart failure, Omland cautions the interpretation of elevated levels in the ICU setting [49]. Our group, along with others, demonstrated that there may be a role in discriminating those patients with from those without cardiac dysfunction in the ICU [50-52]. However, the actual type of cardiac dysfunction is not clarified by the finding of an elevated BNP. Age and gender also must be taken into account in the interpretation of elevated serum levels [53].

**Figure 3:**
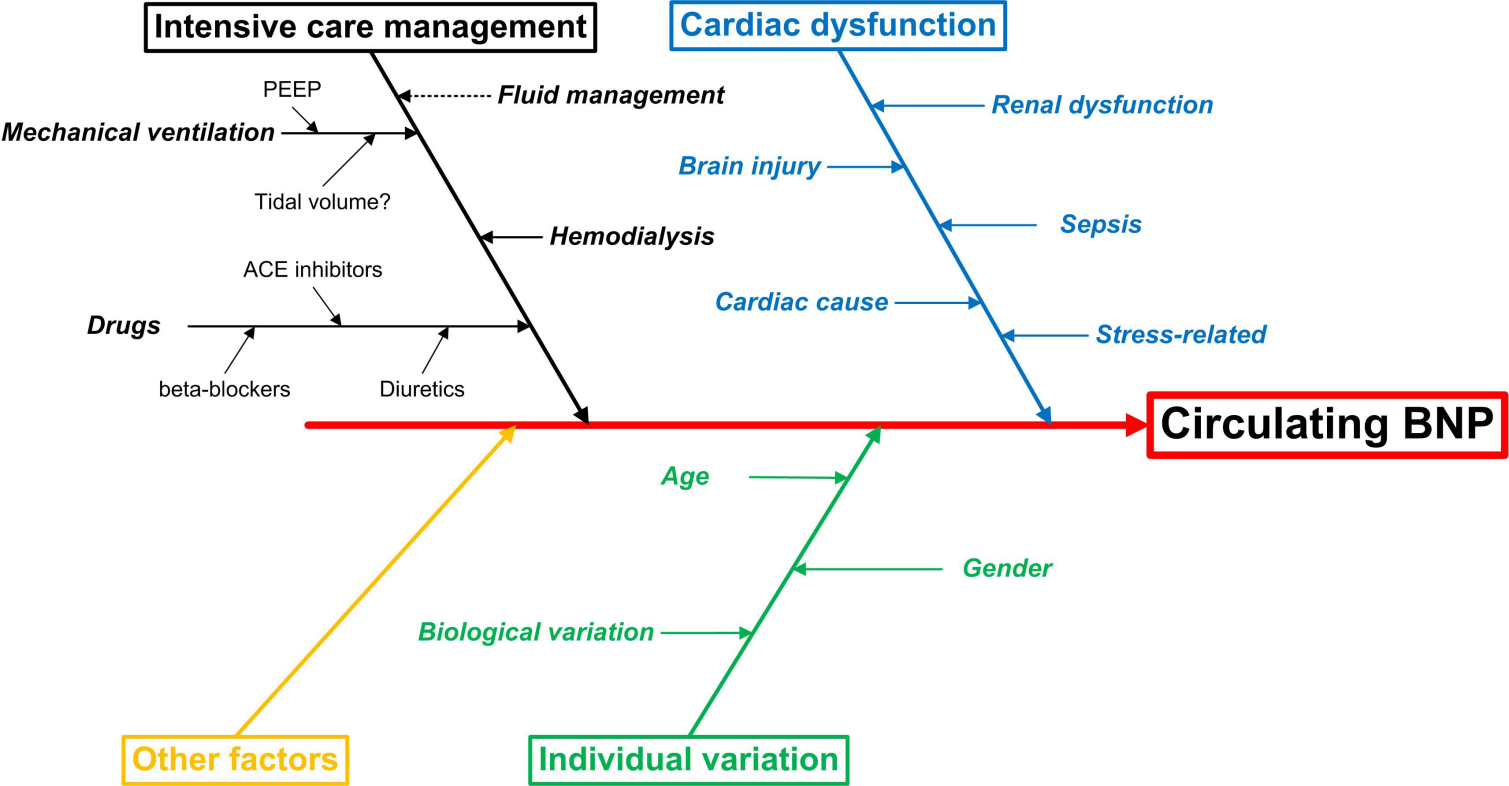
**Contributors to circulating BNP level**.

The challenge is in translating the value of BNP in the diagnosis of HF in the emergency department, or prognostic value of CHF in the outpatient setting, to a role in the critically ill patient. Usually multiple comorbidities, particularly renal failure or sepsis, can cause elevation of serum levels in the absence of heart failure. Numerous factors may contribute to the measurement BNP level in the serum in a single patient (Figure 3).

Does a prognosis role exist for the natriuretic peptides? Numerous studies have sought to clarify a role for NPs as prognostic markers in the ICU setting, yet the results have been conflicting and insufficiently robust to modify a patient's management [54-58]. Although an elevated NP most likely represents cardiac dysfunction even in the critically ill patient, they lack discriminating power to identify what type of cardiac dysfunction is present and cannot be compared with the application of echocardiography, which should be routinely used in critically ill patients with underlying cardiac dysfunction. A in-depth review by Christenson concluded the utilization of BNP or NT-BNP did not have a substantive role in the management of critically ill patients in the ICU [59].

#### Adrenomedullin (ADM)

A peptide of 52 amino acids, ADM is synthesized and found in heart, adrenal medulla, lungs, and kidneys. In the heart, it is produced in response to volume and pressure overload and has similar actions to the NPs [60]. It is a potent vasodilator, inducing diuresis and natriuresis. Plasma ADM levels are significantly elevated in heart failure and AMI, correlated with severity of illness, and have some prognostication for mortality in AMI [61]. It is insufficiently characterized, especially in regard to measurements and clear guidance as to what elevation is important in the ICU setting.

### Usefulness of cardiac biomarkers in noncoronary artery pathologies commonly presenting the in ICU

#### Acute pulmonary embolus

CB can have roles in both the diagnosis and prognosis of a patient with PE. Elevation of cTn results from increased metabolic demand and reduced myocardial perfusion, which causes right ventricular ischemia/microinfarction, whereas increased wall stress results in elevation of BNP. Kucher and Goldhaber in 2003 concluded that a cTn below the cutoff required for that required to diagnose AMI, or a BNP < 50 ng/ml, have a > 98% negative predictive value for in hospital death by PE [62]. La Vecchia and associates demonstrated that a cTnI > 0.06 μg/L was associated with a subsequent mortality of 36% compared with those < 0.06 μg/L, and the former had a greater need for inotropes and mechanical ventilation [63]. The role of NP is less certain, and although some investigators regard NT-proBNP as a better prognosticator than currently used clinical scores in PE, any additional value over cTn or H-FABP in acute PE is not proven [64].

The role of CB in risk stratification is firmly established, and CB are included as principal markers in the European Society of Cardiology guidelines on the diagnosis and management of acute pulmonary embolus [65]. The presence of elevated cTn and/or BNP places the patient outside the low risk category ( < 1% mortality), and when combined with echo features of cardiac dysfunction and hypotension place the patient in the high-risk ( > 15% mortality) category. The combination of specific CB and echocardiographic abnormalities has important management ramifications in regards to consideration of thrombolytic therapy for acute PE, even in the hemodynamically stable patient [62,66].

H-FABP seems to have better diagnostic accuracy and better prognostic value compared with cTn and BNP in cases of PE. Puls and associates in a study of 107 patients found that normal H-FABP on admission predicts an absence of significant complications or death. The PPV for H-FABP was 41% compared with 29% for cTn and 19% for proBNP [67]. In a more recent study involving 101 consecutive patients with confirmed PE and echocardiographic signs of right ventricular overload, but without hypotension or shock, none of the patients with normal H-FABP (N = 87) on admission to the emergency department experienced clinical deterioration, required inotropic support, or died. In the H-FABP-positive group (n = 14), the degree of RV dysfunction was greater, ten had clinical deterioration that required inotropic support, and eight died [68].

#### Sepsis

It has been recognized for more than a decade that elevated cTn in septic patients is associated with a higher mortality [69]. cTn is elevated in 12-85% of ICU patients with sepsis of severe inflammatory syndrome, with a meta-analysis including 3,278 patients from 20 studies, giving a median value of 43% [30].

The wide range of prevalence probably relates to the heterogeneity found in septic studies but clearly an elevated cTn is not uncommon. The real uncertainty comes from the cause of the troponin release, whether it is global myocardial ischemia (i.e., type II AMI), the myocytoxic effects of cytokines, reactive oxygen species or endotoxins, or exacerbation of chronic coronary artery small vessel disease [70]. cTnI also was elevated in septic patients who display evidence of isolated and reversible diastolic dysfunction [71]. Adding to the uncertainty is whether any such myocyte damage is persistent, with autopsy studies not necessarily revealing irreversible damage in shocked patients with premortem elevated cTns [72].

In a study of 207 patients with severe sepsis and septic shock, circulating cTnT was detected in 60% of the patients using the fourth-generation cTnT assays and 100% by the hs-cTnT assay [73]. The levels of hs-cTnT were associated with disease severity and survival, although it was not an independent predictor of in-hospital mortality. The authors proposed a potential role for hs-cTn in sepsis as an early marker of shock. Although NP levels are elevated in sepsis, there is controversy about whether they add to diagnostic or outcome information [54,74].

#### Acute heart failure: non-CAD

cTn is elevated in patients with HF even in the absence of CAD [75,76]. Lacking diagnostic sensitivity and specificity for the diagnosis of heart failure, it is associated with increased risks of mortality and cardiac morbidity [77-79]. For example, elevated cTnI predicts mortality in patients hospitalized for congestive heart failure [75].

#### Renal failure

Because baseline serum cTn levels are already elevated in chronic renal failure (CRF), the diagnosis of a MI is based on > 20% increase in levels at 6-9 hours after presentation [80]. This recommendation by the National Academy of Clinical Biochemistry Laboratory is based on the "older" cTn assays, and the situation has become more challenging with a recent study that demonstrated that in a cohort of asymptomatic patients with end-stage renal disease, 100% had a hs-cTn level > 99% percentile value [81]. In a comparison of the prognostic value of cTn and BNP/NT-proBNP in 143 CRF patients on dialysis, Hickman and associates demonstrated that NT-proBNP was superior to cTn [82].

#### Acute lung injury and weaning

Although classically a "cardiac biomarker," BNP has perhaps best found a role in the ICU in the diagnosis of hypoxic respiratory failure and determining the optimal timing for extubation. Rana and colleagues found that a BNP level < 250 pg/ml supports the diagnosis of acute lung injury (AUC = 0.71), with improved accuracy when patients with renal failure were excluded (AUC = 0.82) [83]. Other studies use varying cutoff serum BNP levels and have different diagnostic accuracy reducing the clinical usefulness of a single sample [84].

A recent study of 100 patients, who underwent a 48-hour spontaneous breathing trial before extubation, underwent echocardiographic evaluation and NP sampling immediately before the trial commenced and at the end, before extubation. A baseline cutoff BNP level of 263 ng/l and 1,343 ng/l for NT-proBNP before the trial, or an increase of 48 and 21 ng/l respectively for BNP and NT-proBNP, accurately predicted those patients who failed extubation as a result of underlying cardiac failure. BNP performed better than NT-proBNP [85]. A prior study recruited patients who received mechanical ventilation for > 24 hours demonstrated that baseline BNP was higher in patients with subsequent weaning failure and correlated to weaning duration [86].

### Multimarker diagnosis of cardiac dysfunction

The use of multiple biomarkers has the theoretical attraction of improving both sensitivity and specificity in the diagnosis of different cardiac disorders, with particular relevance in the ICU where multiple confounding factors exist. The combination of cTnT, ECG, and ischaemia-modified albumin identified ischemia in 95% of patients presenting with chest pain [87]. However, other studies do not support the benefit of multimarkers compared with a single one in the emergency department or ICU [88,89].

In predicting 6-month mortality from acute heart failure, Nunez and associates demonstrated the combination of CA-125 and BNP added prognostic value to that provided by BNP alone [90].

### Potentially future cardiac biomarkers

MicroRNAs (also known as miRs or miRNAs) are approximately 20-25 nucleotide long noncoding ribonucleic acids (RNAs) that negatively regulate or inhibit gene expression by binding to sites in the 3' untranslated region of targeted messenger RNAs [91-94]. They have been found to be involved in almost every biological process, from cellular differentiation and proliferation to cell death and apoptosis. Many different types of miRNAs can be detected in circulating blood, and these miRNAs are present in a remarkably stable form that even withstand repetitive freezing/thawing cycles and are protected against RNases. Of the thousands of miRNAs described to date in humans, many exhibit tissue-specific patterns of expression can be detected in circulating blood. Ones that regulate the cardiovascular system have been identified [95-97]. They can be divided into four groups:

1. miRNAs regulating endothelial function and angiogenesis: miR126, miR17-92 cluster (miR17, miR20a, miR92a), miR130a, miR221, miR21

2. Cardiac myocyte-specific mRNA: miR208a

3. Cardiac myocyte (highly expressed) and skeletal muscle miRNAs: miR1, miR 133a, miR499.

Smooth muscle miRNAs: miR143, miR145

Since the half-life of circulating miRNA is counted in hours and their breakdown do not seem to be compromised by the presence of organ dysfunction, they hold promise as very specific, accurate markers of cardiac dysfunction in the critically ill patient.

## Conclusions

Measuring plasma CB is often a routine undertaking for most critical care physicians, and the increasingly accurate markers developed over the years have an important place in everyday practice. The obvious advantages experienced in the emergency department or cardiology ward are not always shared in the ICU where cofounders frequently make for a more cautious interpretation and careful consideration of subsequent management decisions. Yet, an understanding of the underlying strengths and weaknesses now available from a considerable research and clinical database should lead to an appreciation of their immense value.

## Competing interests

The authors declare that they have no competing interests.

## Authors' contributions

ASM and SJH both drafted manuscript. Both authors read and approved the final manuscript.
